# Effect of Heat Treatment by the Sous-Vide Method on the Quality of Poultry Meat

**DOI:** 10.3390/foods10071610

**Published:** 2021-07-12

**Authors:** Wiesław Przybylski, Danuta Jaworska, Katarzyna Kajak-Siemaszko, Piotr Sałek, Kacper Pakuła

**Affiliations:** Department of Food Gastronomy and Food Hygiene, Institute of Human Nutrition Sciences, Warsaw University of Life Sciences (WULS), Str. Nowoursynowska 166, 02-787 Warsaw, Poland; danuta_jaworska@sggw.edu.pl (D.J.); katarzyna_kajak_siemaszko@sggw.edu.pl (K.K.-S.); piotr_salek@sggw.edu.pl (P.S.); kacper.pakulla@gmail.com (K.P.)

**Keywords:** poultry meat, sous-vide, technological quality, sensory quality

## Abstract

An increase in the consumption of poultry meat has been observed due to its availability, nutritional value, and delicate flavor. These characteristics make it possible to prepare, with the use of spices and other additives, many different dishes and products for increasingly demanding consumers. The sous-vide technique is increasingly being used to give new sensory attributes to dishes in gastronomy. The study aimed to assess the impact of the heat treatment method, i.e., the sous-vide method, as compared to traditional cooking, on the sensory quality of poultry meat, as well as the efficiency of the process with regard to technological quality. The cooking yield with the sous-vide method of processing poultry meat was higher than with the traditional method of cooking in water (88.5% vs. 71.0%, respectively). The meat was also found to be redder (a* = 254 vs. 074) and less yellow (b* = 1512 vs. 1649), as well as more tender. The sensory quality of chicken breast meat obtained by the sous-vide method was higher in terms of attributes such as color tone, tenderness, juiciness, and overall quality. At the same time, it was lower in terms of the odor of cooked meat and the flavor of cooked meat as compared to meat subjected to traditional cooking.

## 1. Introduction

The quantity of poultry produced worldwide is increasing, with production growing by approximately by 3.0% per year. In 2018, the world’s broiler meat production amounted to about 92.7 million metric tons, and is forecasted to increase to about 100 million metric tons by 2021 [1]. This fact is related to a gradual increase in the consumption of poultry meat. In 2020 the consumption of poultry meat increased by 1% worldwide in comparison to 2019 [2]. The increase in the consumption of poultry meat in recent years is associated with its high nutritional value (high level of protein of high biological value, low fat content, and balanced n-6 to n-3 PUFA (polyunsaturated fatty acid) ratio), high level of tenderness, relatively low price, and ease of cooking [3]. The delicate odor and flavor of poultry meat make it possible to prepare, with the use of spices and other additives, a wide variety of dishes and products for increasingly demanding consumers [4]. Important properties of poultry meat also include its low energy and collagen content as well as its delicate structure, thanks to which heat treatment is simple and short, and the prepared meat is easily digestible and tender [4]. In addition, as a rule, the price of poultry meat is relatively low compared to other types of meat. Due to its properties, poultry meat is also recommended by nutritionists for people following an easy-to-digest diet as well as those on a low-calorie dietary regimen [5].

The sous-vide method is gaining in popularity among the many techniques of heat treatment of meat. Historically, this technique has been used since the 1960s, when it was first found to be useful to vacuum-pack products and pasteurize foods in the industry to prolong their shelf life. The method was developed by French chef George Pralus in the mid-1970s [6]. It should be emphasized that this type of cooking in vacuum plastic bags takes place at carefully controlled temperatures and for specific durations with regard to a given food product. Precise temperature control allows better control of the cooking process and texture of the product as compared to when the traditional cooking method is applied [7,8].

There is a growing interest in applying the sous-vide cooking method to various foods to improve consumers’ preferences in the food industry, including food services, catering services, and restaurants. Sous-vide cooking generally uses low temperatures (50–80 °C) for longer periods of time depending upon the type of meat [9]. Additionally, the sous-vide method can be applied to almost all types of foods. When using the sous-vide method, meat is generally cooked for a long period of time at 55–80 °C. At relatively low temperatures the juiciness of meat is maintained, while the flavor and tenderness are improved [10], even in the case of defective meat [11]. An additional advantage of poultry meat is that it is quite easy to prepare for consumption and is “adaptable” to diverse forms of preparation. Meat portions can be vacuum-packed with or without spices and other additives, e.g., vegetables or fruit [12]. The influence of the sous-vide heat treatment method on the technological and sensory qualities of chicken meat is described in the literature [11,12]. However, in these studies the sensory evaluation was carried out to a limited extent. Scaling methods were most often used for evaluation, and the distinguishing attributes of odor were not assessed. Moreover, in the available literature no studies have been found that use advanced sensory methods in poultry meat assessment. For these reasons, the aim of this study was to evaluate the impact of the sous-vide method as compared to traditional cooking on the sensory quality of poultry meat using the Quantitative Descriptive Profile (QDP) method in accordance with ISO 13299: 2016 standards. Moreover, the results were elaborated with multivariate statistical analysis–principal component analysis (PCA). The results of these studies could be useful from a cognitive and practical point of view to optimize the sous-vide method.

## 2. Materials and Methods

### 2.1. Research Material

The research material was comprised of pectoral muscles taken from 20 carcasses of broiler chickens (49-day-old Ross 308 broilers, roosters, bodyweight approximately 2.5 kg). The chickens came from a poultry slaughter plant located in the Mazowieckie Province in Poland. The animals were stunned with CO_2_ and the carcasses were chilled in a shock system (airflow 4 m/s, to a temperature of 2 °C) after exsanguination. The research material was transported from the slaughterhouse to the Department of Gastronomy Technology and Food Hygiene, Warsaw University of Life Sciences WULS–SGGW in polystyrene packaging with inserts ensuring refrigerated transport. The laboratory initially measured the pH (pH_20 h_), color, and natural drip loss.

Then, a preliminary treatment was carried out, consisting of cleaning the raw material and dividing it into portions before thermal treatment. Portions of similar thickness and an average weight of about 250 g were carved from the pectoral muscles. An appropriate amount of raw material was taken gradually from the cold store (4 °C) immediately before the trials.

### 2.2. Research Design

**Figure 1:**
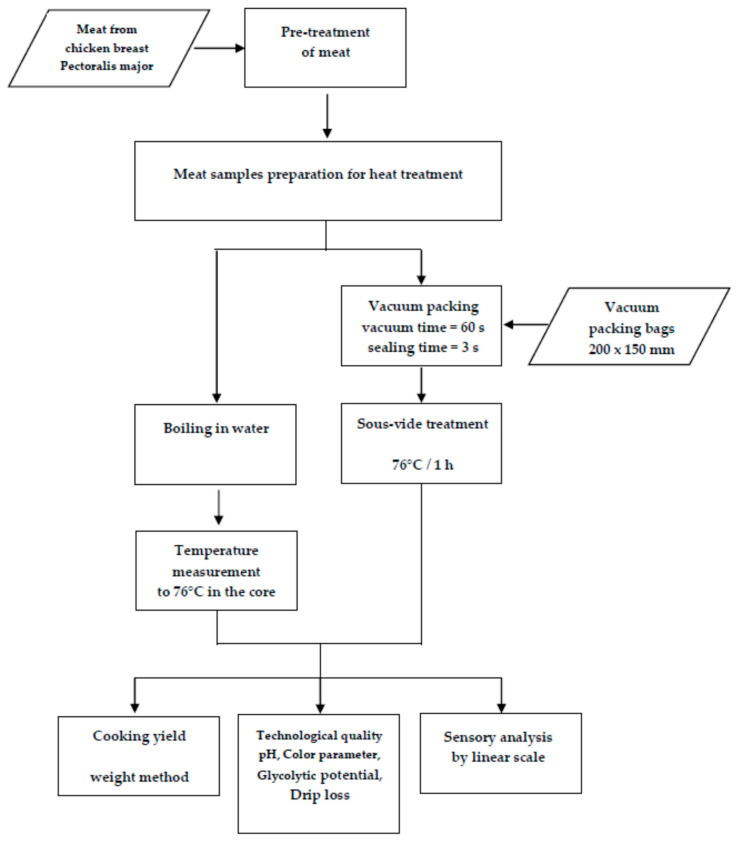
Research scheme.

The research was carried out according to the scheme presented in Figure 1. After pre-treatment (removal of non-edible parts (bones, fat, skin)), the pectoral muscle of the chicken was subjected to proper heat treatment consisting of traditional boiling in water (10 samples) and sous-vide cooking (10 samples). Then, after the heat treatment, the meat was subjected to a sensory evaluation. In addition, calculations were performed to determine the efficiency of both heat treatment processes.

### 2.3. Research Methods

#### 2.3.1. Color Measurement

The measurement of color parameters was carried out using the Konica Minolta CM-2600 spectrophotometer and the CIE Lab (L* a* b*) color system (Konica Minolta^®^ Chroma Meter, Osaka, Japan). Before the measurements, the colorimeter was calibrated on a white and black standard. Measurements were taken before and after the heat treatment at 3 locations on the surface of each meat sample.

#### 2.3.2. The Degree of Acidification of the Muscle (Meat) pH

At 15 min and 20 h after slaughter, the pH value was measured directly in the pectoral muscles (pH_15 min_ and pH_20 h_). The pH value of the muscle (meat) was measured at 3 points, the so-called “heads” of the pectoral muscle (*Pectoralis major*), using a pH meter 330i from WTW^®^ (Weilheim, Germany). The final value for a single muscle was the mean of the 3 measurements. The device was equipped with a dedicated penetrating electrode (SenTix^®^ SP Number 103645) (Weilheim, Germany), designed to measure the pH directly in meat and meat products.

#### 2.3.3. Glucose and Lactate Concentration in Tissue, and Muscle Glycolytic Potential

Glucose and lactate were measured in drip loss by using an Accu-Chek Active^®^ glucometer (Accu-Chek Sensor Comfort^®^, Roche, Germany), as described by Przybylski et al. [13]. The muscle glycolytic potential (PG) was calculated according to the formula *PG = (2 × glucose) + lactate* adapted from the work of Monin and Sellier [14], and was expressed as millimoles (mmol) of lactate.

#### 2.3.4. Heat Treatment of Meat

##### Boiling in Water

The chicken meat was cooked in a pot with a diameter of 18 cm. The water in the pot was heated on a 3.5 kW induction cooker manufactured by Stalgast (Warsaw, Poland). The cooking of each meat sample was performed at a temperature of about 100 °C using 750 mL of unsalted water. Individual samples were cooked individually. The test material was put into boiling water and then cooked and covered until the temperature in the geometric center of the meat samples reached 76 °C for 2 min. The temperature was measured with a TME thermometer (HENDI Polska Sp. z o.o., Warsaw, Poland) to measure the processing time.

##### Sous-Vide Cooking

The raw material was vacuum-packed in Hendi bags, which are intended for the preparation of sous-vide dishes and have 2 layers: an inner layer of 60 μm made of polyethylene intended for contact with food, and an outer layer of 15 μm made of polyamide that increases durability and guarantees air-tightness. These bags have a total thickness of 75 μm, can be used at temperatures from −20 °C to +110 °C, and are suitable for chamber vacuum packaging machines. The dimensions of the bags were 20 × 15 cm (HENDI Polska Sp. z o.o., Warsaw, Poland). The bags were made of plastic approved for contact with food. The tested meat samples were individually packed. Packing was carried out using a vacuum packing machine of the Stalgast (Warsaw, Poland) chamber type. For each meat sample, the time of packing (60 s) and sealing (3 s) was identical.

The sous-vide low-temperature treatment process was carried out in a Hendi low-temperature cooking unit (HENDI Polska Sp. z o.o., Warsaw, Poland). There were 20 L of water in the device. The meat samples were cooked at 76 °C for 60 min, and the time taken for temperature equilibration was measured.

After heat treatment, the breast meat was cooled to room temperature (20 °C) and weighed to evaluate the efficiency of the cooking process.

#### 2.3.5. Cooking Yield

The cooking yield was used to determine the efficiency of both heat treatment processes. The cooking yield was calculated as the ratio of the weight of the meat sample before the treatment to the weight after the treatment. The measurement results are shown as a percentage. The precision (0.01 g) electronic balance WTB 2000 apparatus from RADWAG (Radom, Poland) was used in the study.

#### 2.3.6. Determination of the Cutting Force

Determination of the cutting force was done using the ZWICKI 1120 apparatus (ZwickRoell GmbH & Co. KG, Ulm, Germany). From the heat-treated meat samples, samples with a cross-section of 1 × 1 cm with a longitudinal arrangement of muscle fibers were cut. During the test, the maximum force of cutting of the meat sample with a Warner-Bratzler attachment (equipped with a flat knife) and the depth of penetration of the knife at which the maximum force occurred were measured. An initial force was 0.5 N and the test speed was 50 mm/min. A measuring head with a range of 2–1000 N was used.

#### 2.3.7. Assessment of Sensory Quality

The sensory quality of the tested meat was determined after heat treatment and cooling with the use of Quantitative Descriptive Profiles (QDP)-enabling qualitative and quantitative determination of the sensory characteristics of a given product [15]. A linear scale (100 mm) converted to numerical values (0–10 c.u.) was used and expressed as conventional units. Each attribute’s scale had marked anchors, as presented in Table 1.

In the preliminary session, attributes were selected and defined as follows: 4 attributes of odor (cooked meat, sour, fatty, other), 2 attributes of color (the tone of the meat color, homogeneity of color), and 2 texture attributes (tenderness, juiciness). In addition, 6 flavor attributes were selected (cooked meat, sour, fatty, salty, bitter, other) (Table 1).

The meat samples were portioned into 3–4 mm thick slices, and 2 slices were placed into disposable boxes made of clear food-safe plastic. The boxes were then closed and individually coded with 3-digit codes.

The sensory evaluation was performed by a panel of 10 trained assessors of sensory evaluation. The panel had many years of experience in evaluating meat and meat products according to the ISO 8586:2012 procedure [16]. The meat samples given in random order were assessed in 2 independent sessions. Because the sessions were performed in duplicate, a total of 20 unit scores were obtained. The evaluation conditions were determined according to Baryłko-Pikielna and Matuszewska [17].

#### 2.3.8. Statistical Analysis

The basic descriptive statistics (mean, standard deviation) were calculated. The normality of the distribution of data was checked using the Shapiro–Wilk test. The significance of differences between the groups in terms of the analyzed methods of heat treatment (boiled vs. sous-vide) was determined using the Student’s *t*-test. The significance of differences was estimated with the statistical significance coefficient *p* < 0.05. Principal component analysis (PCA) was performed for the results obtained in the QDP method of sensory analysis. Pearson’s linear correlation coefficients between the variables determining the sensory quality were calculated. The obtained data were developed using STATISTICA version 13.3 software (TIBCO Software Inc. 2017, Palo Alto, CA, USA, Statistica data analysis software system, version 13, http://statistica.io, accessed on 28 May 2021).

## 3. Results

The quality characteristics of the raw material intended for testing are presented in Table 2. The data that characterized the quality of poultry meat selected for heat treatment using traditional cooking and sous-vide methods did not differ significantly (*t*-test, *p* < 0.05).

Table 3 shows the characteristics of meat assessed based on cooking yield and color parameters, and the results of the instrumental cutting test after heat treatment using two methods.

The statistical analysis showed significant differences in cooking yield after heat treatment, in the color parameters a* and b*, and in instrumentally established tenderness expressed as the depth of the maximum cutting force. Poultry meat after sous-vide treatment was characterized by a significantly higher cooking yield (by 17.5%). The poultry meat was redder (higher value of the a* color parameter) and less yellow (lower value of the b* color parameter). The meat was also more tender (Table 3).

Many significant differences between the tested methods of heat treatment were obtained following QDP (Table 4). Significant differences in odor were obtained in the case of such characteristics as the odor of cooked meat, sourness, and the attribute defined as “other”. Moreover, the meat differed significantly in the tone of color, tenderness, juiciness, and the taste of cooked meat as well as salty and “other” tastes (Table 4).

**Figure 2:**
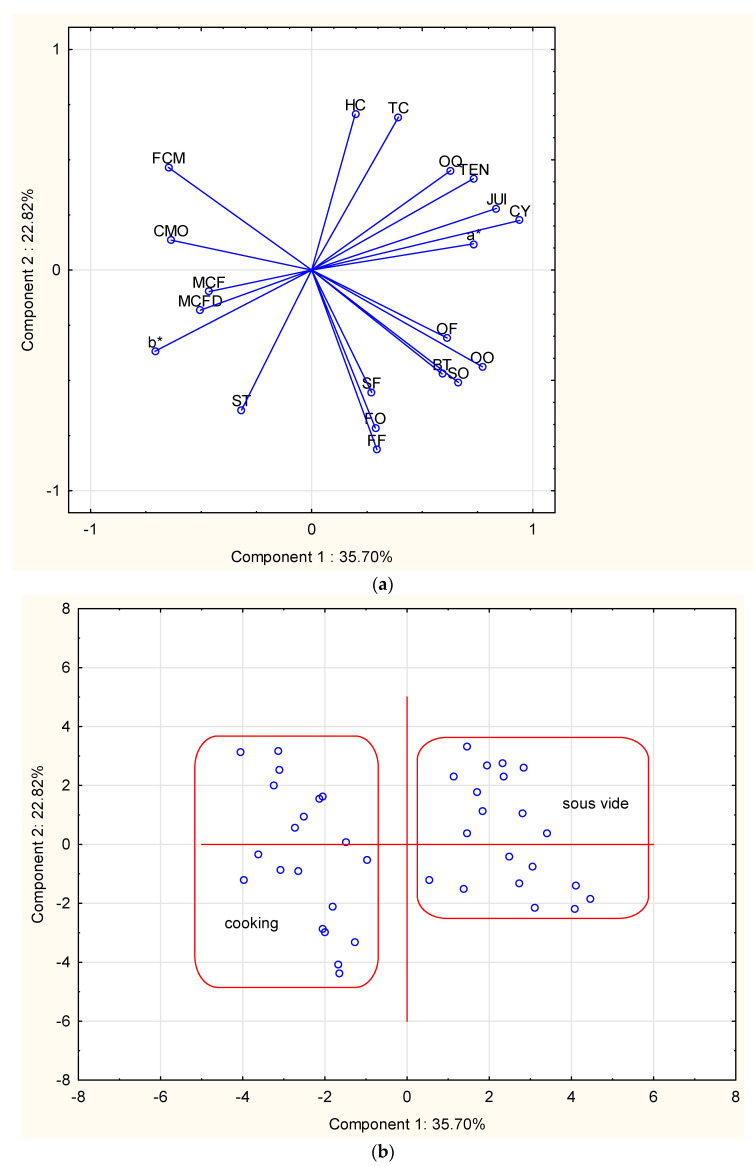
Results of principal component analysis (PCA). (**a**) projection of variables on the plane of PC; HC: homogeneity of color; TC: tone of color; OQ: overall quality; TEN: tenderness; JUI: juiciness; CY: cooking yield; a*:redness of meat: OF: other flavor; OO: other odor; BT: bitter taste; SO: sour odor; SF: sour flavor; FO: fatty odor; FF: fatty flavor; ST: salty taste; b*: yellowness of meat; MCFD: maximum cutting force depth; MCF: maximum cutting force; CMO: cooked meat odor; FCM: flavor of cooked meat; (**b**) projection of cases on the plane of PC; Samples from individual methods are included in the indicated areas.

The mentioned characteristics of the sensory quality profile ultimately resulted in significant differences in the overall sensory quality of the poultry meat. The sous-vide meat was more tender and was juicier, and its overall sensory quality was rated higher by the sensory evaluation team (Table 2). At the same time, this meat was distinguished by a less intense odor of cooked meat and had a sourer odor. The panelist emphasized that it was also distinguished by “other” odor notes (Figure 2). The results of a different sensory and instrumentally assessed quality profile were reflected in the results of principal components analysis (PCA) (Figure 2). More than 58% of the total variability in the area of sensory quality was explained by the first two main components. The first component was most strongly related to the characteristics of tenderness, juiciness, cooking yield, and redness. Meanwhile, the second component was mostly related to attributes for the evaluation of sensory color and fatty odor and flavor (Figure 2a). This relationship separated the tested material into two different groups, differentiated by the type of heat treatment (Figure 2b). With regard to the taste attributes, it was found that it was distinguished by a lower intense flavor of cooked meat and a less salty taste. At the same time, the panelists more often emphasized “other” flavor notes (Table 4). However, in the final evaluation the overall quality was rated as significantly higher (*p* < 0.001).

The correlation coefficients among the sensory quality attributes are presented in Table 5. The tenderness and juiciness of the meat had the greatest share in the overall meat quality (respectively r = 0.61 and 0.77 at *p* < 0.05). Moreover, the overall quality was positively influenced by the color attributes—its tone and homogeneity (respectively r = 0.43 and 0.41 at *p* < 0.05). The cooked flavor was positively correlated with the cooked odor (r = 0.54 *p* < 0.05) and negatively correlated with a sour and “other” odor (respectively: r = −0.68 and r = −0.72 *p* < 0.05). There was also a positive correlation in the case of the “other” odor and the sour and fatty flavor (respectively r = 0.38 and 0.51 *p* < 0.05), and a negative correlation with flavor of cooked meat (r = −0.72 *p* < 0.05). The sour flavor of meat was positively correlated with the sour odor (r = 0.48 *p* < 0.05), and negatively correlated with the odor (r = −0.44 *p* < 0.05) and flavor (r = −0.49 *p* < 0.05) cooked meat. The fatty flavor was positively correlated with the fatty odor.

## 4. Discussion

When meat is heated to a temperature above 70 °C, myoglobin is completely denatured, as a result of which the color of the meat changes. However, the change depends on its type, as it is different in the case of breast meat and leg meat [18]. Poultry meats cooked at low temperatures often have pink color defection, affecting appearance and causing consumer complaints concerning the impression of uncooked or bloody meat [19]. Hong et al. [20] reported that many consumers were not satisfied with the pink color of cold-cooked poultry meat, as they believed it indicated bloody and undercooked meat, and thus feared risk. These phenomena have led to the limited study of sous-vide cooked chicken breast compared to other sous-vide cooked vegetables, fish, and meat products. Additionally, the choice of packaging material in the sous-vide method is of fundamental importance, as research shows that the degree of oxygen penetration (i.e., DOP) through the packaging significantly affects the quality and durability of the stored meat. The degree of DOP penetration through the packaging material is important for the sensory evaluation of color, as well as storage period and the proportion of red color (a*). The intensity of the a* parameter of cooked meat is inversely related to the degree of denaturation of myoglobin, a process that takes place between 55 and 65 °C and continues up to 75 or 80 °C. Sous-vide cooked meat is lighter and redder in color than meat cooked by conventional cooking, which can be explained by the changes in the myoglobin pigment during cooking [21].

Based on the results of the research and comparison of the efficiency of the traditional cooking method and the sous-vide method, it is clear that the sous-vide method allows for a higher cooking yield. As Soletska et al. [22] observed, chicken samples cooked using the sous-vide method show a four-fold reduction in losses as compared to the traditional techniques, while the product yield is also increased by 19%. Moreover, Michalak-Majewska et al. and Ruiz-Carrascal et al. [23,24] also indicated that the sous-vide method results in lower losses in the volume and weight of prepared dishes (only around 10%). The yield depends mainly on the temperature of the heat treatment process and the duration of the treatment. If meat is exposed to high temperatures for a long time, it becomes dry, which leads to a lower product weight and consequently a lower yield [25,26]. According to Park et al. [26], increasing the temperature from 60 to 70 °C increased the losses by 11–12%, and extending the time from 1 h to 3 h increased the losses by 6–8%.

During conventional cooking weight losses occur; the degree of loss depends on the heating temperature and the type of raw material. These losses for chicken meat can vary between 24% and 32%. In addition to the loss of weight during the cooking of meat, the leakage of a liquid containing mainly water but also nutrients changes the chemical composition of the meat [27]. Due to sous-vide cooking, the products retain vitamins, dyes, and unsaturated fatty acids that do not dissolve in water [21]. A retention of small water-soluble nutrients was noted by Botinestean et al. [28]. Research by Ramanea et al. [29] showed that while cooking poultry fillets using the sous-vide method there is a slight loss of soluble proteins, with these losses being twice as high in the case of cooked meat portions with the addition of vegetables and fruit as compared to the case of meat cooked without such additives.

In addition, during cooking a specific taste of cooked meat is produced as a result of protein denaturation and changes in nitrogen extract fractions. The taste of cooked meat is determined by nitrogen ring compounds and derivatives of furfurol, thiophene, and thiolate. These compounds are the result of transformations of sulfur amino acids at a temperature of ca. 70 °C. The taste of cooked meat appears even with gentle heat treatment (at a temperature of 50 °C) and is mainly related to the hydrolysis of proteins and the transformation of nitrogen extracts. As the temperature rises, the flavor of cooked meat fluctuates [30]. In cooked meat, the proportion of protein and fat increases by more than 50%, while the content of water and minerals (meat juice) that pass into drip loss decreases. The passage of nutrients into the decoction is a disadvantage of the cooking process in water.

Baldwin [7] confirmed that cooking in heat-stable vacuum bags improves meat shelf life and flavor. An extended shelf-life of products thanks to the sous-vide method by eliminating the possibility of recontamination, changes in moisture, oxidation, or loss of aroma was also shown by [29]. Unlike the characteristics of tenderness and juiciness, a cooked meat flavor (which is mainly attributed to the volatile aromatic compounds) commonly develops at temperatures above 70 °C, and thus sous-vide meat cooked at a lower temperature exhibits poorer flavor than meat cooked at a higher temperature because of the extent of Maillard reaction [31].

As shown by calculated correlation coefficients between the assessed determinants of sensory quality, the overall sensory quality was mostly related to the tenderness and juiciness of meat after heat treatment. Similarly, in studies by Górska et al. [25] the overall sensory quality of pork was significantly related to its tenderness and juiciness. The results presented in Table 3 and Table 4 clearly show that in terms of cooking yield and texture characteristics, such as tenderness or juiciness, meat samples after the sous-vide process were more highly assessed.

The most important textural effect of heat treatment is the tenderization of meat, which occurs as a result of the transformation (thermohydrolysis) of collagen at a temperature of 60–70 °C. Temperature is the main factor in reducing collagen-induced hardness. Since poultry meat contains procollagen, which is an immature form of collagen which swells easily in the aqueous environment, poultry meat does not need to undergo long and intensive thermal treatment. The temperature for the denaturing of collagen protein has been reported to range from 53 to 63 °C [32], while for myofibrillar proteins (mainly myosin) denaturation occurs at 40–60 °C, with subsequent gelation of collagen fiber at 60–70 °C followed by denaturation of actin at 70–80 °C [33]. Generally, both the cooking temperature and time affect meat tenderness, and increased cooking temperature has a greater effect on fiber shrinkage than increased cooking time. Consequently, lower temperature in sous-vide cooking results in tender meat, and sensory juiciness also increases as the cooking temperature and time are reduced [34]. Data in the literature data confirm that the tenderness and juiciness of sous-vide chicken breast meat were rated higher [26]. Thanks to the use of low temperature, the structure of the meat remains intact as its fibers do not shrink quickly, and the prepared product is juicy and has a higher nutritional value due to the better digestibility of proteins. Moreover, an effect of the low-temperature heating process is a change of collagen into gelatin, which gives the meat a more appreciated structure [35]. Denaturation of muscle proteins and a significant shrinkage in diameter and length of fibers occur above 60 °C [36]. The tenderization achieved through sous-vide cooking is mainly attributed to the reduced denaturation of proteins at the typically lower temperatures used, the weakening of connective tissue through collagen solubilization, and retention of water [22].

Church and Parson [37] also emphasize a significant difference between the quality of meat prepared using the sous-vide method and the quality of meat prepared using other methods. As a result of the research, it was found that poultry meat prepared with the sous-vide method had a more intense flavor and juiciness than the meat cooked using the traditional method. According to McGee [38], a sous-vide treated product will have the same temperature throughout its volume. This kind of effect cannot be achieved by traditional methods in which the outer layer of the product is subjected to high temperatures. In this regard, a frying process produces a product that is fried on the outside, while the inside may be raw. Traditional methods result in differences in the juiciness of different parts of the product, e.g., a fried chicken breast will be less juicy on top compared to the inside. The sous-vide method ensures the same degree of juiciness throughout the entire section of the meat portion.

The juiciness of meat is a very important feature of meat and significantly affects the satisfaction of consumers who, as a rule, do not like meat that is not very juicy. Meat juiciness is also influenced by the method of heat treatment, particularly its temperature and time [25]. According to Baldwin [7], lean poultry meat remains juicy if it is processed at a temperature not exceeding 60–65 °C. Furthermore, McGee [39] claims that poultry meat has adequate juiciness and tenderness if the heat treatment temperature does not exceed 65 °C in the geometric center of the meat, and the use of higher temperatures is possible in the case of processing meat with higher fat content. Based on the results of the sensory evaluation, it can be concluded that the sous-vide meat samples obtained a better rating compared to the samples cooked using the traditional method, which means that the low-temperature thermal treatment method has a positive effect on the sensory quality of poultry. With the increased process parameters the hardness and the intensity of the cooked meat or fish flavor increased, while the juiciness and off-flavor perception decreased [8]. The results of our research showing that the tenderness and juiciness of the meat had the greatest share in the overall quality are confirmed by the research of Thompson et al. [39], who found that features such as tenderness, juiciness, taste and general quality are strongly correlated with each other. Hong et al. [20] also emphasize the importance of tenderness, which is a very important attribute of cooked meat and strongly influences consumer preferences. As has already been mentioned, the texture of the meat is one of its most important features and is shaped during the heat treatment process. According to Hong et al. [20], sous-vide processing can improve the sensory quality of chicken breast fillets, preventing a defective product in the form of dry and disintegrating meat. While muscle fibers begin to contract at 35–40 °C and this process continues up to 80 °C, low-temperature treatment leads to soft and juicy chicken breast meat. Precise temperature control in sous-vide cooking allows the control of both fast and slow changes, and thus the quality of products prepared using the sous-vide method is significantly different from that of those processed using other methods [7].

## 5. Conclusions

The cooking yield using the sous-vide method of processing poultry meat was distinctly higher than with the traditional method of cooking in water (88.5 vs. 71.0, respectively). The meat was also redder and less yellow, as well as more tender. The sensory quality of chicken breast meat obtained by the sous-vide method was higher in terms of features such as color tone, tenderness, juiciness, and overall quality. At the same time, it was lower in terms of the odor of cooked meat and the flavor of cooked meat as compared to meat subjected to traditional cooking. The overall quality of the meat was positively correlated with its tenderness (r = 0.61) and juiciness (r = 0.77), and to a lower extent with the tone of color (r = 0.43) and homogeneity of color (r = 0.41). Based on the obtained results regarding the sensory quality (mainly flavor), further studies should be undertaken on the use of spices to improve the flavor characteristics of meat subjected to sous-vide treatment.

## Figures and Tables

**Table 1:** Sensory attributes of the quality of chicken breast meat.

Sensory Attribute	The Marks of Anchors
Odor	
Cooked meat	No intensity–High intensity
Sour	No intensity–High intensity
Fatty	No intensity–High intensity
“Other”	No intensity–High intensity
Color	
Tone of color	Light beige–Dark beige
Homogeneity of color	Homogenous–Heterogenous
Texture	
Tenderness	Hard–Very tender, soft
Juiciness	No juicy–Very juicy
Flavor	
Cooked meat	No intensity–High intensity
Sour	No intensity–High intensity
Fatty	No intensity–High intensity
Salty taste	No intensity–High intensity
Bitter taste	No intensity–High intensity
“Other” (e.g., off flavor)	No intensity–High intensity
Overall quality	Low–Very high

**Table 2:** Quality characteristics of raw meat samples (n = 20).

Feature	Heat Treatment Method		Significance Level
	Cooking	Sous-Vide	*p*
pH_15 min_	6.75 ± 0.17	6.70 ± 0.11	0.432
pH_20 h_	5.98 ± 0.13	5.96 ± 0.12	0.654
Glucose (mmol/L)	26.06 ± 7.09	25.31 ± 7.07	0.755
Lactate (mmol/L)	98.73 ± 20.17	95.88 ± 19.49	0.710
Glycolytic Potential (mmol/L)	152.23 ± 25.65	147.14 ± 26.56	0.613
Color parameters: L*	54.11 ± 2.13	53.07 ± 1.93	0.267
a*	0.36 ± 1.00	0.08 ± 0.83	0.506
b*	10.17 ± 0.88	9.55 ± 1.45	0.266
Drip loss 24 h	0.53 ± 0.34	0.49 ± 0.15	0.743
Drip loss 48 h	0.86 ± 0.49	0.86 ± 0.17	0.964
Drip loss 72 h	1.23 ± 0.64	1.32 ± 0.43	0.711

**Table 3:** Characteristics of meat after heat treatment (n = 20).

Feature	Heat Treatment Method		Significance Level
	Cooking	Sous-Vide	*p*
Cooking yield (%)	71.01 ^A^ ± 2.64	88.48 ^B^ ± 1.94	0.001
Color parameters: L*	83.73 ± 1.29	84.26 ± 0.63	0.263
a*	0.74 ^A^ ± 0.64	2.54 ^B^ ± 0.69	0.001
b*	16.49 ^A^ ± 1.02	15.12 ^B^ ± 0.51	0.001
Maximum cutting force depth (mm)	5.95 ^A^ ± 0.46	5.18 ^B^ ± 0.54	0.002
Maximum cutting force (N)	20.69 ± 4.95	19.68 ± 3.52	0.615

^A,B^ indicate that values denoted by different superscript letters are statistically different (*p* < 0.05).

**Table 4:** Characteristics of sensory quality of meat after heat treatment (0–10 c.u.), n = 20.

Feature	Heat Treatment Method		Significance Level
	Cooking	Sous-Vide	*p*
Odor			
Cooked meat	8.33 ^A^ ± 0.34	7.79 ^B^ ± 0.3	0.001
Sour	1.21 ^A^ ± 0.37	1.54 ^B^ ± 0.18	0.020
Fatty	1.25 ± 0.39	1.33 ± 0.26	0.582
“Other”	0.97 ^A^ ± 0.40	1.73 ^B^ ± 0.52	0.002
Color			
Tone	8.70 ^A^ ± 0.32	9.03 ^B^ ± 0.31	0.032
Homogeneity	8.36 ± 0.60	8.74 ± 0.33	0.104
Texture			
Tenderness	8.26 ^A^ ±0.50	8.92 ^B^ ± 0.33	0.002
Juiciness	7.26 ^A^ ± 0.66	8.94 ^B^ ± 0.42	0.001
Flavor			
Cooked meat	8.50 ^A^ ± 0.30	8.05 ^B^ ± 0.27	0.002
Sour	1.12 ± 0.27	1.29 ± 0.32	0.195
Fatty	1.10 ± 0.27	1.15 ± 0.29	0.713
Salty taste	1.25 ^A^ ± 0.23	1.04 ^B^ ± 0.16	0.026
Bitter taste	0.78 ± 0.17	1.00 ± 0.33	0.075
“Other” (e.g., off flavor)	1.02 ^A^ ± 0.32	1.63 ^B^ ± 0.80	0.039
Overall quality	7.81 ^A^ ± 0.31	8.31 ^B^ ± 0.19	0.001

^A,B^ indicate that values denoted by different superscript letters are statistically different (*p* < 0.05).

**Table 5:** Correlation coefficients between the tested sensory quality traits.

Attributes	Correlation Coefficients														
	Odor				Color		Texture		Flavor						
	Cooked Meat	Sour	Fatty	“Other”	Tone	Homogeneity	Tenderness	Juiciness	Cooked Meat	Sour	Fatty	Salty Taste	Bitter Taste	“Other”	Overall Quality
Odor															
Cooked meat		−0.45 *	−0.16	−0.59 *	0.05	0.12	−0.37 *	−0.51 *	0.54 *	−0.44 *	−0.18	0.32 *	−0.11	−0.23	−0.26
Sour			0.51 *	0.83 *	−0.05	−0.17	0.25	0.34 *	−0.68 *	0.48 *	0.56 *	0.11	0.54 *	0.34 *	0.18
Fatty				0.51 *	−0.21	−0.35 *	−0.07	0.00	−0.38 *	0.20	0.85 *	0.33 *	0.58 *	0.42 *	−0.13
“Other”					−0.01	−0.10	0.41 *	0.55 *	−0.72 *	0.38 *	0.51 *	0.07	0.60 *	0.54 *	0.25
Color															
Tone						0.75 *	0.60 *	0.41 *	0.06	−0.47 *	−0.34 *	−0.45 *	0.11	0.12	0.43 *
Homogeneity							0.36 *	0.32 *	0.19	−0.43 *	−0.43 *	−0.34 *	−0.01	0.03	0.41 *
Texture															
Tenderness								0.79 *	−0.25	−0.02	−0.13	−0.51 *	0.27	0.39 *	0.61 *
Juiciness									−0.37 *	0.09	0.00	−0.36 *	0.38 *	0.41 *	0.77 *
Flavor															
Cooked meat										−0.49 *	−0.39 *	−0.01	−0.57 *	−0.54 *	−0.05
Sour											0.36 *	0.16	0.24	0.21	0.03
Fatty									-			0.51 *	0.65 *	0.47 *	−0.08
Salty taste													0.27	−0.01	−0.28
Bitter taste														0.76 *	0.08
“Other” flavor												-			0.11

* correlation coefficient significant at *p* < 0.05.

## Data Availability

The data presented in this article is available on reasonable request, from the corresponding author.
